# A Leaky Noisy-OR Bayesian Network Applied to Genetic Counseling in Dogs

**DOI:** 10.3390/ani10061104

**Published:** 2020-06-26

**Authors:** Johann. C. Detilleux

**Affiliations:** Fundamental and Applied Research in Animal Health (FARAH), Veterinary Faculty, University of Liege, Quartier Vallée 2, 6 Avenue de Cureghem, 4000 Liège, Belgium; jdetilleux@uliege.be; Tel.: +32-4-3664215

**Keywords:** disease control, animal, prevention, decision support, genetics

## Abstract

**Simple Summary:**

Genetic disorders represent a serious health problem for companion animals and combating such disorders is a real challenge. Bayes networks facilitate the objective assessment of the risk of such disorders. We apply the methodology to answer two typical questions in genetic counselling, i.e., the risk for an animal of showing clinical signs of a genetic disease when the result at the genetic test is known and the risk of testing positive for the mutant allele when the genetic test is not made. Results showed the network is appropriate to answer objectively and transparently both questions under a variety of alternative scenarios. It can be updated automatically and can be represented visually so interactive discussion are easy between the veterinarian and his/her interlocutor.

**Abstract:**

Genetic disorders are very frequent in dogs but evaluating individualized risks of their occurrence can be uncertain. Bayesian networks are tools to characterize and analyze such events. The paper illustrates their benefits and challenges in answering two typical questions in genetic counselling: (1) What is the probability of a test-positive animal showing clinical signs of the disease? (2) What is the risk of testing positive for the mutant allele when one parent presents clinical signs? Current limited knowledge on the hereditary mode of transmission of degenerative myelopathy and on the effects of sex, diet, exercise regimen and age on the occurrence of clinical signs concurrent with the finding of the deleterious mutation was retrieved from the scientific literature. Uncertainty on this information was converted into prior Beta distributions and leaky-noisy OR models were used to construct the conditional probability tables necessary to answer the questions. Results showed the network is appropriate to answer objectively and transparently both questions under a variety of scenarios. Once users of the network have agreed with its structure and the values of the priors, computations are straightforward. The network can be updated automatically and can be represented visually so interactive discussion are easy between the veterinarian and his/her interlocutor.

## 1. Introduction

Genetic disorders are very frequent in domestic animals and combating such disorders is a real challenge [1,2]. As an example among many others, 23% of mutant alleles were observed in the gene coding for the superoxide dismutase 1 (SOD1) in a population of Bernese Mountain dogs that had a hip radiograph taken in Belgium, the Netherlands or Germany [3]. A mutation in this gene has been associated with degenerative myelopathy (DM) in several breeds but not in others in which clinical signs of DM have yet go be reported [4,5]. Inversely, animals that tested positive at the genetic test may not present associated clinical signs during their lifetime [6]. Additionally, a dog breeder may not be sure an animal presenting clinical signs will transmit the unfavorable SOD1 allele to its offspring.

Clinical signs specific to DM include an initial upper motor neuron spastic paraparesis and general proprioceptive ataxia in the pelvic limbs progressing to a flaccid lower motor neuron tetraparesis [7]. Along with this weakness, urinary tract infections, pressure sores and anxiety may appear. Diseases may mimic DM signs like hip dysplasia, intervertebral disc disease myelitis, degenerative lumbosacral stenosis, spinal cord neoplasia and certain parasitic diseases which cause inflammation of the spinal cord [8].

In such situations of uncertainty, the accuracy with which veterinarians evaluate treatment and preventive measures or advise dog breeders is not perfect. They often draw on their observations, practical experience and expertise which may not be understood or shared by the interlocutor. They may also take advantage of tools designed to improve clinical decision-making such as Bayesian networks (BN). Indeed, BN are tools for representing and integrating prior knowledge, observations and reasoning in a structure of probabilistic relationships [9]. More specifically, a BN is a probabilistic graphical model that can be depicted as a directed acyclic graph made up of nodes and arcs. Each node represents a variable and the arcs indicate how one variable (i.e., possible cause) directly influences another (i.e., effect). Each node is associated with a conditional probability table (CPT) that reflects the probability of each instance contained within the node. These probabilities may be obtained from expert knowledge (e.g., scientific literature) or learned from data [10].

The number of elements in a CPT grows exponentially with the number of arcs which makes its construction time consuming and often impossible with certainty [11]. Fortunately, in probabilistic gate models, the number of elements grows linearly instead of exponentially which simplifies the construction of the CPT [12]. The most popular of these models are the Noisy-OR model and, its extension, called the Leaky Noisy-OR (LN_NOR) model (e.g., [13]). In a LN_NOR, all variables can be either {T} (i.e., true or present) or {F} (i.e., false or absent). The model is “noisy” in the sense that a variable X_i_ can produce the effect Y with some “link” probability p_i_ such that:

p_i_ = pr(Y = {T} | X_i_ = {T} and all other variables = {F})

where “all other variables” include modeled and non-modeled variables. Modeled variables (X_i_) are those known to influence Y and non-modeled variables are the others, assuming it is often impossible to capture all the possible causes for an effect. Then, the “leak” or “background” probability is:

p_0_ = pr(Y = {T} | X_i_ = {F} for i = 1, 2, …n).

It is the probability that the effect is {T} when the variable X_i_ is {F} and corresponds to the fact that variables other than X_i_ may exist and can produce the effect Y. Then, one constructs the CPT by computing pr(Y = {T}| X_p_), here called “risk”, where X_p_ is a subset of variables X_i_ that are {T}. Diez [13] showed:

pr(Y = {T}| X_p_) = 1 − (1 − p_0_) ∏_i_ (1 − p_i_), and

pr(Y = {F}| X_p_) = (1 − p_0_) ∏_i_ (1 − p_i_)

for i = 1, 2, …, m_p_ where m_p_ is the number of variables in X_p_.

Because they are not known with certainty, a degree of belief is assigned to the “leak” and “link” probabilities. This can be done with prior Beta distributions because these distributions are conjugates of Bernoulli distributions which are specific for probabilities and proportions defined in the range 0–1. Beta distributions can take a very wide variety of shapes that are defined with only two parameters [14]. The “informativeness” of the priors for these parameters vary from being “informative” at one end to being “non-informative” at the other end [15]. A prior is “informative” if it expresses specific, definite information about the parameter. It is “non-informative” when it reflects a balance among all possible values of the parameters.

The objective of this paper is to use a LN_NOR to evaluate (1) the risk for an animal to show clinical signs associated with DM when the result at the SOD1 genetic test is known and (2) the risk of testing positive at the SOD1 genetic test when one parent presents clinical signs associated with DM. Clinical signs include manifestations specific and concomitant to DM, including coexistent diseases. The objective is not intended to provide a predictive tool for all DM dogs because the output of the LN_NOR is a function of the amount and type of information shared between veterinarians and their interlocutors. The DM is used as a running example and the procedure can be adapted to other diseases.

## 2. Materials and Methods

The first step consisted in searching the literature to determine which and how genetic and non-genetic factors may influence the apparition of clinical signs associated with DM. The second one consisted in constructing the network relating individuals in the net and the third one in constructing the CPT.

### 2.1. Step 1: Identification of Potential Risk Factors

In DM, dogs with the defective genetic variant(s) may develop clinical signs but not all will do so [6,8]. Two mutations have been found in SOD1: the mutant *SOD1:c.118A* allele is widespread and common among dogs with DM whereas the *SOD1:c.52T* allele is rare and appears to be limited to Bernese Mountain dogs [16]. A modifier gene coding for a SP110 nuclear body protein (involved in the regulation of gene transcription) has also been associated with an increased probability of developing signs of DM, and an earlier age of onset in Pembroke Welsh Corgis (but not in Boxer) homozygous for the mutation [17]. These findings suggest results at the genetic test should not be over-emphasized and should be considered as only one factor in the diagnosis process [18].

Age, diet, and exercise have been identified as potential non-genetic factors affecting the probability to show signs of progressive weakness in the back legs. Indeed, clinical signs of DM appear usually between 8 and 14 years of age, with a progression over time to a complete inability to walk 6 months to 2 years after the onset of symptoms [4]. Owners of a DM dog often elect for euthanasia within one year after onset of clinical signs [5]. Although onset of DM is not affected by exercise and diet, they may improve the survival time in dogs with a presumptive diagnosis of DM [19]. Lack of leg co-ordination may exacerbate signs of intervertebral disc disease and hip dysplasia which often coexist with DM [7,20] and are influenced by diet and exercise. For example, exercise maintenance of optimum body weight is known to be effective for reducing the signs associated with dysplasia and arthritis [21]. Excessive adiposity may result in excessive mechanical loading that worsens clinical signs in patients with arthritis and generalized systemic inflammation propagated by obesity contribute to neurologic signs associated with intervertebral disc disease [22]. Note that it is not precisely described in the literature how these potential non-genetic risk factors may influence quantitatively the risk of clinical signs associated with DM.

### 2.2. Step 2: Setting Up the Network

**Figure 1:**
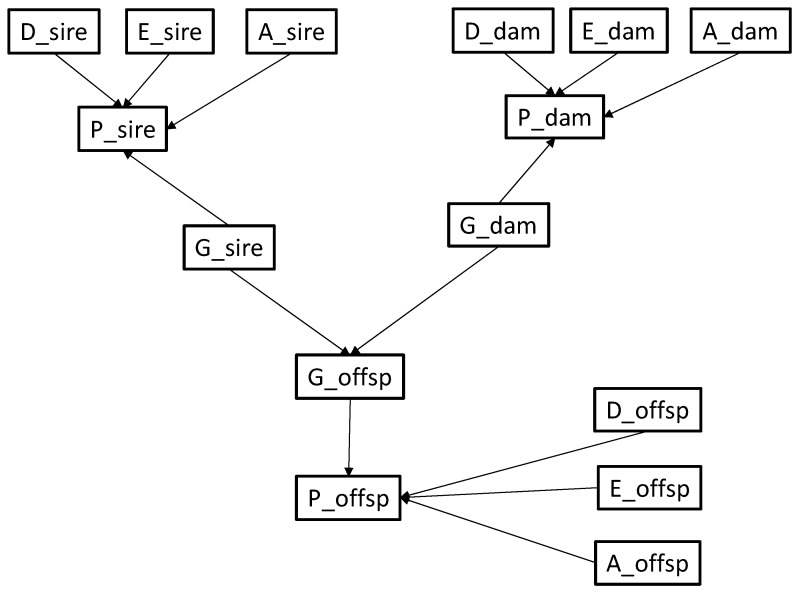
Representation of the nodes and arcs of the network.

The Netica “6.03”^®^ software (e.g., [23]) was used to construct the network taking into account that genetics, diet, exercise and age (but not sex) may all influence clinical signs of DM as identified in step 1. As a start, the BN is purposely simple with only 3 individuals (Figure 1). The central part displays the relationships between the nodes representing the genotypes of the parents (G_sire and G_dam) and of their offspring (G_offsp) at the disease locus. The homozygous genotypes are denoted AA and BB for the wild (A) and mutated (B) alleles, respectively. The heterozygous genotype is denoted AB. When nothing is known, at start, equal probabilities (i.e., 1/3) are given to the AA, BB and AB genotypes of the parents (i.e., no breed risk term is included). One may also consider that disease allele frequency is low in the general population and is different across breeds [24].

For each animal, the remaining nodes symbolize the phenotype, age, diet, and level of exercise. For example, for a particular sire, nodes representing its diet (D_sire), exercise level (E_sire), age range (A_sire) and genotype (G_sire) are connected to its phenotype (P_sire). The arcs are similar for all individuals in the net.

### 2.3. Step 3: Constructing the Conditional Probability Tables

Four CPTs were created: three to associate predisposing factors with the presence of clinical signs associated with DM (one per individual) and one to associate the genotypes of the parents with the genotype of their offspring. For this last CPT, Mendelian laws of inheritance were followed. For the others, LN-NOR models were devised. According to the terminology of LN-NOR models, variable Y = {T} when clinical DM is observed and Y = {F} otherwise. Variables X_i_ are the modeled risk factors: genetics (i = 1), diet (i = 2), exercise (i = 3), and age (i = 4) with X_i_ = {T} if the factor is associated with Y and X_i_ = {F} otherwise. Then, probabilities are as follows:

- ‘leak’ is the probability of a dog to present clinical signs if all modeled risk factors are {F}: pr(Y = {T} | X_i_ = {F} for i = 1, 2, 3, 4),
- ‘link NG1’ is the probability of a dog to present clinical signs if one non-genetic risk factor is {T}, i.e., either the diet is inadequate, or the level of exercise is disproportionate, or the animal is within the age range with the highest susceptibility to present clinical signs: pr(Y = {T} | X_i_ = {T} for i = 2 or i = 3 or i = 4),
- ‘link G’ is the probability of a dog to present clinical signs if it is homozygous for the defective allele: pr(Y = {T} | X_1_ = {T}),
- ‘risk NG2’ is the probability of a dog to present clinical signs if two non-genetic risk factors are {T}: pr(Y = {T} | X_i_ = {T} and X_j_ = {T} for i, j = 2, 3, 4 and i ≠ j),
- ‘risk NG3’ is the probability of a dog to present clinical signs if three non-genetic risk factors are {T}: pr(Y = {T} | X_i_ = {T} and X_j_ = {T} and X_k_ = {T} for i, j, k = 2, 3, 4, i ≠ j and j ≠ k),
- ‘risk NG1+G’, ‘risk NG2+G’, ‘risk NG3+G’ are the probabilities of a dog to present clinical signs if it is homozygous for the defective allele and one, two or three non-genetic risk factors are {T}, respectively.

**Figure 2:**
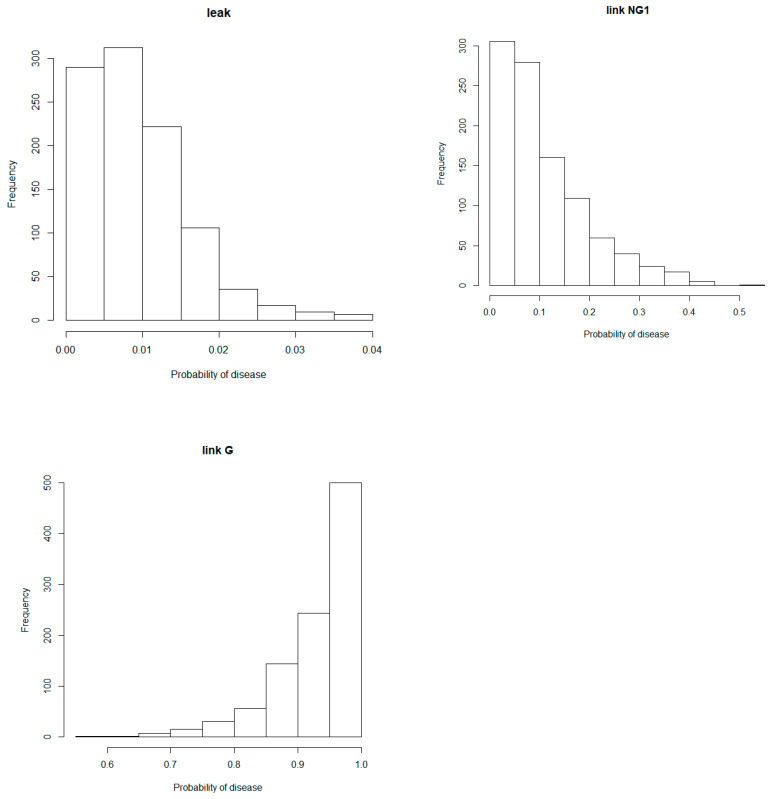
Beta distributions when the mean is set at 1% (leak), 10% (link NG1) and 90% (link G).

At this stage, the task remains to assign values for the “leak”, “link NG1” and “link G” probabilities so that all other probabilities can be estimated according to the LN_OR model. A flexible tool is to assign values sampled from Beta distributions because these distributions represent all the possible values of a probability (from 0 to 1) when we do not know the exact value of that probability. They take on many different shapes according to the values of two parameters. In our settings, these parameters determine the range that the “leak” and “link” probabilities will take and should be assessed on a case-by-case basis. These parameters represent the uncertainties identified in Step 1. Here, for illustrative purposes, seven combinations of parameters (Table 1) were created in R [25]: Means of ‘link NG1’ were set as identical (10%, 30% or 50%) for all non-genetic risk factors and lower than or equal to means of ‘link G’ (10%, 50% or 90%). The mean of ‘leak’ was set at 1% in all 7 combinations. In Figure 2, Beta distributions when the means are set at 1%, 10% and 90% are given. Notice the Beta distributions for ‘link NG1’ is right-skewed with most values close to null and below 10% illustrating the lack of precise information on the effects of the non-genetic risk factors on the probability for an animal to shown clinical signs.

Necessary programming codes in R and Netica^®^ software can be found in the Supplementary Materials.

## 3. Results

The first question to be answered was about the probabilities of showing clinical signs associated with DM when the result at the genetic test is known. These probabilities may vary whether or not one, two or three non-genetic risk factors are present, as shown in Table 1 for each combination of the parameters of the Beta distributions. From these, one can observe probabilities are higher when the dog is tested homozygous recessive than otherwise (e.g., ‘risk NG2+G’ is higher than ‘risk NG2’) and increase with the number of non-genetic risk factors (e.g., ‘risk NG3’ is higher than ‘risk NG2’) whatever the group of prior values. Let us consider for example, the case where it is believed that the probability to present clinical signs associated with DM is on average 10% when the diet or the level of exercise is inadequate and on average 90% when the result at the genetic test is positive (animal homozygous for the defective allele). Then, on average, the risk for a dog to present clinical signs when both its diet and level of exercise are inadequate is 19.04% and 94.05% when the result at the test is negative or positive, respectively.

**Figure 3:**
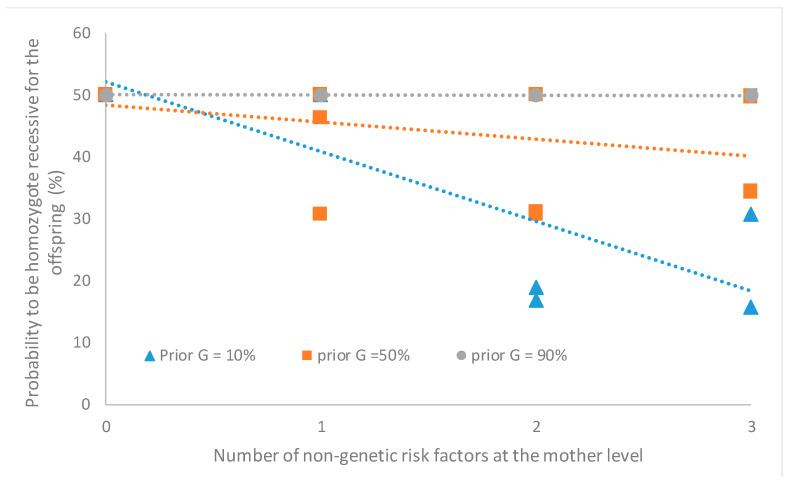
Probability to be homozygous for the deleterious allele for the offspring as a function of the number of non-genetic risk factors of degenerative myelopathy observed at the mother level.

The second question to be answered was about the probability of testing positive for the mutant allele when the genetic test is not performed. This can be evaluated for different mating. For example, one may know the mother presents clinical signs but have no information about the father. In such case, probabilities for the offspring to be AA, AB or BB are 0%, 50% and 50%, respectively, when the age, diet and level of exercise of the mother are {F}. But probabilities for the offspring to be BB decrease when the number of non-genetic factors in the {T} class increases, especially when the prior values for the genetic factor are low (Figure 3). If, in another mating, both parents are clinically healthy, probabilities of the offspring to be AA, AB or BB are 56.25%, 37.5% and 6.25%, respectively, when age, diet and level of exercise of both parents are {F}. If not, the probability to be BB increases up to 6.42% when the number of non-genetic risk factors in the {T} class increase.

Probabilities for the offspring to be BB knowing its mother present clinical signs vary also with the frequency of the mutant allele in the population. It is 50% when the allelic frequency is 50% and close to 10% when the allelic frequency is 10% (close of the values observed in populations of Pembroke Welsh Corgis and Boxers [24] and assuming Hardy Weinberg equilibrium).

## 4. Discussion

The contribution of this paper is to illustrate the benefits of BN and LN-OR models in providing decision support to veterinarians when answering two typical questions of genetic counseling. Such models can be helpful because the elimination from the gene pool of all dogs testing positive for a deleterious mutation can lead to a genetic bottleneck and inbreeding depression. In addition, one reviewer pointed out that “this particular model could be very useful in a cross-breeding population since genetic testing is less uniform in mixed-breed mating”.

In its present form, the net structure is deliberately simple to allow hand-checking computations and a good understanding of the benefits. However, assumptions can be released as more information on the disease become available and tailored to the questions that need to be answered (e.g., considering the prevalence of DM in a particular breed). As stated above, the goal is not to provide a predictive tool for all DM dogs especially that manifestation of DM signs is still poorly understood.

One benefit of BN is linked to the transparency of these models in the sense that outcomes can be easily interpreted on the basis of the input parameters. As an example, in this study, it makes sense that estimated probabilities to present clinical signs increase when the number of non-genetic risk factors increased (Table 1). It also makes sense that these probabilities are higher when the animal is tested homozygous recessive. It was also expected to observe a decrease in the probabilities of the offspring to be homozygote recessive with an increase in the number of non-genetic risk factors associated with the presence of clinical signs in the mother (Figure 3). Given their transparency, it is straightforward to verify and debug BN models and to discuss different alternatives with all interested parties. Veterinarians and their interlocutors may even modify input parameters in a participatory approach.

A second benefit is that uncertainty can be handled through the use of prior probability distributions that span the spectrum of possibilities which is not possible with standard models (e.g., linear or logistic regression) in which explanatory variables are fixed at specific values. Here, different prior Beta distributions were proposed (Figure 2, Table 1) which can be automatically updated to incorporate new knowledge [26]. Indeed, results state the SOD1 tests are helpful but must be interpreted with care. The probability of presenting clinical signs increases when results at the test are positive but this does not mean that all positive dogs will present clinical signs. If the veterinarian and interlocutor are confident in a specific value for the probability to show clinical signs associated with DM, they may change interactively the values of the parameters of the Beta distribution for the “link G” and the risk estimate will be updated automatically. The parameters of the Beta distribution determine the range that the “leak” and “link” probabilities could take and should be assessed on a case-by-case basis.

Uncertainty could also be reduced by incorporating information on a greater number of relatives in the network. To do so, it is enough to add new nodes representing the genotypes or phenotypes of the relatives and the links between these new nodes. This added information could be particularly useful when genetic testing is too expensive, when it can only be performed in a limited number of laboratories or when it may involve significant time delay. One can also easily update the model to incorporate information about the sensitivity and specificity of the genetic test in determining the real genetic status of the animal. Indeed, analytical validity has been shown to vary across laboratories [27]. To do so, it is enough to add a ‘test’ node to represent the result at the test (e.g., positive or negative) and a link between this ‘test’ node and the node representing the true genotype of the individual. After such additional information is added, risk estimates are automatically updated which allows interactive exchanges between BN users [28].

**Figure 4:**
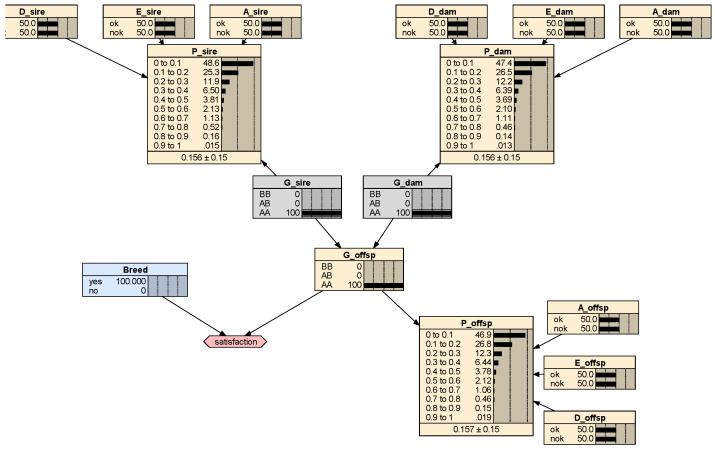
Screen shot of the network on Netica^®^ with decision node (blue rectangle entitled ‘breed’) and utility node (six-sided figure entitled ‘satisfaction’).

A third benefit is that BN can easily be enhanced with decision nodes that represent decisions to be made on the basis of their utility, i.e., a cardinal value that reflects the perceived preference for a particular decision outcome [29]. Utilities should be determined by the dog owner or breeder to reflect the own private value they put into the animal [30]. In Netica^®^, decision and utility nodes are simply added to the BN (Figure 4). As an example, a dog owner may set utilities for the genotype AA, AB and BB at 100, 100 and 0 to reflect the preference for healthy individuals. In such cases, mating two individuals homozygous for the wild allele would lead to the maximal utility of 100 while mating two healthy individuals for which no test is done (no knowledge of their genotypic status) will lead to an utility of 93.69. Alternatively, a breeder may put more emphasis on the genotypic status of the animals and set utilities at 100, 50 and 0 for the genotypes AA, AB and BB, respectively. In this case, the utility of mating two clinically healthy parents is only 74.88, lower than the maximum of 100. This illustrates how trade-offs can be evaluated in a personal manner by veterinarians and their interlocutors: in the example, the dog owner may contest the necessity of performing genetic tests (because utilities of 93.69 is close to 100) while the breeder may be more convinced of it (because utility is 74.88, lower than 100).

Bayesian network can also be used backward. As an example, one can estimate the genotypic status of the parents when one has information on their offspring: if the offspring is tested BB, probabilities are 66.7%, 33.3% and 0% for each parent to be BB, AB and AA, respectively. If the offspring is clinically ill (unknown genotype), these probabilities will vary according to the prior values given to the non-genetic risk factors. For example, and given the model assumptions, the probability for each parent to be BB is 100% when age, diet and level of exercise of the offspring are {F} and 66.5% when all are {T} (prior values for Beta distributions of 10% for ‘link NG1’ and 90% for ‘link G’).

Obviously, BN models also have limitations. An important one is the necessity to categorize predisposing factors in two states ({T} and {F}) which may not completely capture the complexity of the effect. However, it may be more suitable in practical settings because dog owners and breeders may find it difficult to express their knowledge about non-genetic risk factors in a more complex form. It is also possible to create multiple and independent ‘two state’ categories to capture the complete range of effects of a predisposing factor. Another limitation is that feedback-loop effects cannot be included in a BN but this is less essential here as the direction of the relationship between risk factors and phenotype is one-way.

## 5. Conclusions

The BN and LN_OR models proposed in this paper were applied to answer questions about the risk of a genetic disease in dogs. Even though the structure of the net was kept simple, it evaluated objectively and transparently the risk based upon information available in the scientific literature and/or result at a genetic test. The net can be easily updated with incoming new information and could serve as a basis to answer questions about other genetic disorders.

Even though the article contains mathematical equations underlying the BN and LN_OR models, it is possible to present purely visual representations of the network so it can be understood by people who would normally balk at mathematical equations so interactive discussions are made easier between the veterinarian and his/her interlocutor.

## Figures and Tables

**Table 1:** Mean (st. deviation) values (%) for the distributions of probabilities (link and risk) to present clinical signs when one, two or three non-genetic (NG1, NG2, NG3) and genetic (G) risk factors are associated with the disease.

	Group 1	Group 2	Group 3	Group 4	Group 5	Group 6	Group 7
NG1	10 (9.49)	30 (23.39)	10 (9.49)	30 (23.39)	10 (9.49)	30 (23.39)	50 (29.02)
NG2	17.57 (14.43)	49.90 (28.75)	19.14 (15.44)	48.49 (28.62)	19.04 (15.04)	50.82 (28.56)	60.37 (39.53)
NG3	23.88 (18.67)	59.70 (29.72)	25.87 (19.51)	58.29 (29.85)	25.81 (19.18)	60.72 (29.59)	65.25 (39.18)
G	10 (9.49)	10 (9.49)	50 (29.02)	50 (29.02)	90 (6.04)	90 (6.04)	50 (29.02)
NG1 + G	18.60 (10.99)	40.48 (22.12)	56.64 (26.26)	64.92 (23.76)	93.47 (6.03)	95.51 (4.88)	76.09 (30.32)
NG2 + G	25.39 (14.96)	55.09 (26.26)	60.40 (24.91)	73.35 (22.47)	94.05 (5.64)	96.67 (4.18)	80.81 (28.55)
NG3 + G	31.11 (18.28)	63.90 (27.00)	63.70 (24.06)	78.46 (21.26)	94.54 (5.35)	97.37 (3.74)	83.22 (27.38)

## Supplementary Materials

The following are available online at https://www.mdpi.com/2076-2615/10/6/1104/s1, File: Codes for constructing the network.
