# Supplementary material for: A Leaky Noisy-OR Bayesian Network Applied to Genetic Counseling in Dogs
Source: Animals (Basel). 2020 Jun 26;10(6):1104. doi: 10.3390/ani10061104 (PMC7341277; doi:10.3390/ani10061104)
Supplement: Supplementary file 1 [file animals-10-01104-s001.pdf]

Supporting information

Codes for constructing the network.

1/ R codes to compute parameters for the Beta distributions with mean value ‘init = 0.5’ and p(0.001< init < 0.999)=0.95 (adapted from the codes provided by T. Radkte : https://minimizeregret.com/post/2016/10/01/setting-hyperparameters-of-a-beta-distribution/)

```
x<-seq(0,1,length=1000)
init<-0.5
min<-0.001
max<-0.999
ww<-optimHyperpars(init,min,max)
alpha<-ww$optimalParameters$alpha
beta<-ww$optimalParameters$beta

optimHyperpars <- function(m,l,u, prob = 0.95, maxESS = 300, maxAlpha = 100)
 {
  maxAlpha <- round(ifelse(m*maxESS < maxAlpha, m*maxESS, maxAlpha))
   computeDeviation <- function(a)
   {
   b <- round(a*(1-m)/m)
   betaIntegral <- pbeta(u, a, b) - pbeta(l, a, b)
   deviation <- (prob - betaIntegral)^2
   return(c(beta = b, deviation = deviation, coverage = betaIntegral))
```

```
  }
 optimSet <- 1:maxAlpha
 parameterSet <- cbind(alpha = optimSet,
                       plyr::ldply(optimSet, computeDeviation))
 optimalParameters <- parameterSet[which.min(parameterSet$deviation),1:2]
 optimalDeviation <- min(parameterSet$deviation)
 uniqueMinimum <- min(
   parameterSet$deviation[-which.min(parameterSet$deviation)]) !=
   optimalDeviation

 if (!uniqueMinimum) message("The minimum is not unique.")

 return(list(optimalParameters = optimalParameters,
             optimalDeviation = optimalDeviation,
             uniqueMinimum = uniqueMinimum,
             parameterSet = parameterSet))
}
```

2/ Parameters alpha and beta were obtained for each combination of risk factors. Example of the R code necessary to compute ‘risk NG2’ :

```
leak<-rbeta(x, 1,100)
start<-rbeta(x,1,9)
p<-1-((1-leak)*(1-start)*(1-start))
hist(p,main="risk NG2",xlab="Probability of disease")
```

```
qts<-quantile(p,probs=c(.025,.975))

mm<-mean(p)

ww<-optimHyperpars(mm,qts[1],qts[2])

alpha<-ww$optimalParameters$alpha

beta<-ww$optimalParameters$beta
```

3/ Building of the conditional probability tables (CPT) that associate predisposing factors and presence of clinical signs based on the values of alpha and beta obtained for the corresponding Beta distributions. Example of the Netica code for the CPT of a sire.

```
p(P_sire | D_sire, E_sire, A_sire, G_sire) =

 (E_sire==ok && D_sire==ok && A_sire==ok && G_sire==AB) ?BetaDist(P_sire, 1,100):

 (E_sire==ok && D_sire==ok && A_sire==ok && G_sire== AA) ?BetaDist(P_sire, 1,100):

 (E_sire==nok && D_sire==ok && A_sire==ok && G_sire==AB) ? BetaDist(P_sire, 2,16) :

 (E_sire==ok && D_sire==nok && A_sire==ok && G_sire==AB) ? BetaDist(P_sire, 2,16) :

 (E_sire==ok && D_sire==ok && A_sire==nok && G_sire==AB) ? BetaDist(P_sire, 2,16) :

 (E_sire==nok && D_sire==ok && A_sire==ok && G_sire== AA) ? BetaDist(P_sire, 2,16) :

 (E_sire==ok && D_sire==nok && A_sire==ok && G_sire== AA) ? BetaDist(P_sire, 2,16) :

 (E_sire==ok && D_sire==ok && A_sire==nok && G_sire== AA) ? BetaDist(P_sire, 2,16) :

 (E_sire==ok && D_sire==ok && A_sire==ok && G_sire==BB) ? BetaDist(P_sire, 2,16) :

 (E_sire==nok && D_sire==nok && A_sire==ok && G_sire== AA) ? BetaDist(P_sire, 1,4) :

 (E_sire==nok && D_sire==ok && A_sire==nok && G_sire== AA) ? BetaDist(P_sire, 1,4)

:

 (E_sire==ok && D_sire==nok && A_sire==nok && G_sire== AA) ? BetaDist(P_sire, 1,4)

:
```

```
(E_sire==nok && D_sire==nok && A_sire==ok && G_sire==AB) ? BetaDist(P_sire, 1,4) :
(E_sire==nok && D_sire==ok && A_sire==nok && G_sire==AB) ? BetaDist(P_sire, 1,4)  :
(E_sire==ok && D_sire==nok && A_sire==nok && G_sire==AB) ? BetaDist(P_sire, 1,4)  :
(E_sire==nok && D_sire==ok && A_sire==ok && G_sire== BB) ? BetaDist(P_sire, 3,12) :
(E_sire==ok && D_sire==nok && A_sire==ok && G_sire== BB) ? BetaDist(P_sire, 3,12) :
(E_sire==ok && D_sire==ok && A_sire==nok && G_sire== BB) ? BetaDist(P_sire, 3,12) :
(E_sire==nok && D_sire==nok && A_sire==nok && G_sire==AA) ? BetaDist(P_sire, 1,3)
:
(E_sire==nok && D_sire==nok && A_sire==nok && G_sire==AB) ? BetaDist(P_sire, 1,3)
:
(E_sire==nok && D_sire==ok && A_sire==nok && G_sire== BB) ? BetaDist(P_sire, 2,5) :
(E_sire==ok && D_sire==nok && A_sire==nok && G_sire== BB) ? BetaDist(P_sire, 2,5):
(E_sire==nok && D_sire==nok && A_sire==ok && G_sire== BB) ? BetaDist(P_sire, 2,5):
BetaDist(P_sire, 2,4)
```
